# Perforators Detected in Computed Tomography Angiography for Anterolateral Thigh Free Flap: Am I the Only One Who Feels Inaccurate?

**DOI:** 10.3390/jcm12124139

**Published:** 2023-06-20

**Authors:** Hyounmin Kim, In-ho Cha, Hyung Jun Kim, Woong Nam, Hyunwoo Yang, Gibum Shin, Chena Lee, Dongwook Kim

**Affiliations:** 1 Department of Oral & Maxillofacial Surgery, Yonsei University College of Dentistry, Seoul 03722, Republic of Korea; netizen93@yuhs.ac (H.K.); cha8764@yuhs.ac (I.-h.C.); kimoms@yuhs.ac (H.J.K.); omsnam@yuhs.ac (W.N.); baachooo@yuhs.ac (H.Y.); shkbm12@yuhs.ac (G.S.); 2 Department of Oral and Maxillofacial Radiology, Yonsei University College of Dentistry, Seoul 03722, Republic of Korea; chenalee@yuhs.ac

**Keywords:** perforator flap, computed tomography angiography, ultrasonography Doppler

## Abstract

Background: The number, location, and pattern of perforators in anterolateral thigh(ALT) flap vary and predicting them preoperatively will aid in reconstructing complex head and neck defects. This article suggests guidelines for utilizing CTA imagery to predict perforators of ALT-free flaps. Methods: We retrospectively analyzed 53 Korean patients who underwent reconstruction with ALT flap in our department from March 2021 to July 2022. The location, course, origin, and pedicle lengths predicted in CTA and confirmed in the operation field were recorded and compared. Results: Among the 85 intraoperatively-found perforators, 79 were also identified in CTA. Six perforators unidentified in CTA were newly found intraoperatively. The positive predictive value of CTA for the perforator was 100%, with a sensitivity of 79/85 = 92.9%. Of the 79 perforators depicted by the CTA for the flap, CTA and intraoperative findings for the course were consistent in 52 cases, a 9.6 mm median discrepancy being noted between the actual location and CTA. Conclusions: The overall pattern or location of perforation was not significantly different between the two, although some differences were observed. It is suggested that the addition of Doppler imaging, in conjunction with CTA, can aid in perforator detection and help minimize such discrepancies.

## 1. Introduction

**Figure 1:**
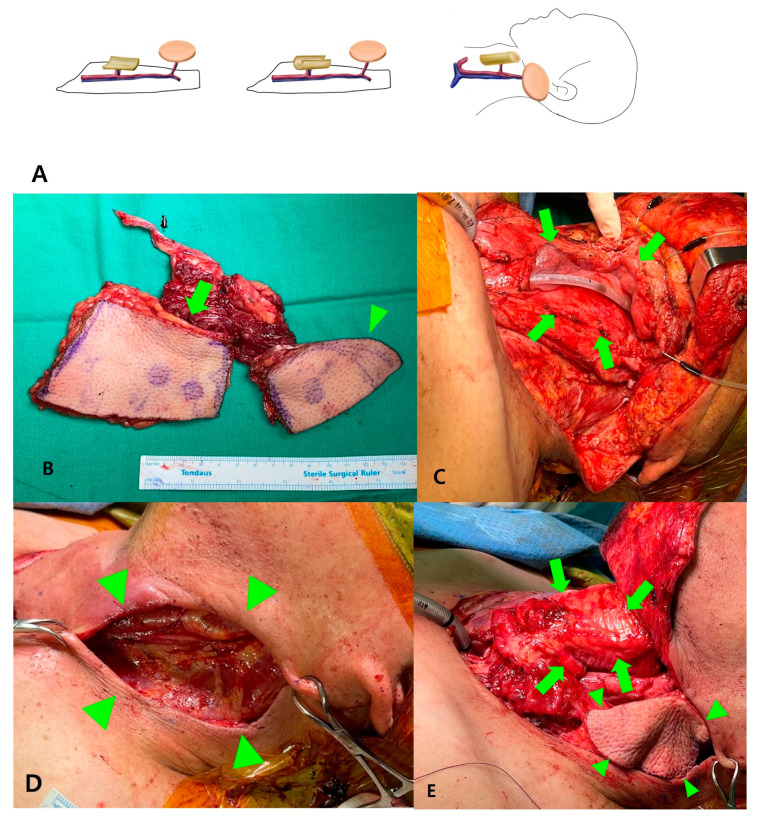
Chimeric ALT for reconstructing pharynx and overlying neck skin. (**A**) Complete separation of two skin paddle is mandatory for reconstructing two defects apart with single ALT. (**B**) Perforator based chimeric ALT is harvested. Note that two skin paddles, one for neopharynx (arrow) and another for neck skin (arrowhead) are completely separated based on the perforators of each. (**C**) Defect after pharyngectomy (arrow) (**D**) and excision of involved overlying skin (arrowhead). (**E**) After flap insetting. The larger skin paddle is rolled to form neopharynx (arrow), and the smaller skin paddle is located at the skin defect (arrowhead).

Though the ALT flap obtained its popularity as a “freestyle flap”, this does not necessarily mean that it can be designed and configured freely, without any restrictions [1]. For instance, when the chimeric design is required to reconstruct composite defects such as pharynx and overlying neck skin, (Figure 1) a surgeon knowing the number and configuration of perforators preoperatively can optimize design and outcome [1,2,3,4]. Knowing the length of a vascular pedicle preoperatively in reconstructing maxillary defects will let a surgeon know if it is long enough to reach neck vessels to be anastomosed, or if vein grafts should be prepared, especially in vessel-depleted settings. To overcome such limitations, efforts to visualize the location of perforators and reproduce them in patients have been reported. These studies have used a handheld Doppler device, computed tomography angiography (CTA), maximum intensity projection (MIP) reconstruction, or color Doppler ultrasound (CDU) [5,6]. These studies have shown success in identifying the location of the perforators with great accuracy through various preoperative imaging modalities and reproducing during surgery [7]. However, these devices are limited by the need for a radiologist to read the images, and the inability to instantly localize the patients at the operation table. Given these limitations, the authors evaluated the utility of predicting the location of the perforator using CTA alone in 53 anterolateral thigh flap recipients and comparing it to the actual location.

## 2. Materials and Methods

We retrospectively analyzed 53 Korean patients who had undergone ALT free flap reconstruction in the Department of Oral and Maxillofacial Surgery, from March 2021 to July 2022. All patients underwent multidetector CTA of lower extremity before surgery. CT scanners (Siemens Somatom Definition Flash (FLASH), FORCE, or X.cite Siemens Healthineers, Revolution CT, GE Healthcare, Chicago, IL, USA) were used for preoperative perforator mapping, with a slide thickness of 2 mm.

A single surgeon (D.K.) preoperatively recorded the point of anterior superior iliac spine (ASIS), superolateral point of patella on CTA, as well as the location and type of course of perforators shown on CTA. The perforators found in the CTA were also recorded preoperatively. The point of branching from the femoral artery and the point perforating from the septum or muscle were recorded to predict whether it was a septocutaneous or musculocutaneous type, and to predict the length of the pedicle.

During the operation, after visualizing the perforator and confirming with a handheld Doppler, the location, type, and course of the perforators were recorded. Type of recipient vessels were also recorded.

The objective features of the enrolled patients, the location and type of perforators, etc. were organized using descriptive statistics. The correlation between the location and pedicle length was tested by Kruskal–Willis test. Statistical analysis was performed using the R programming language (R Core Team, Vienna, Austria, 2022). Differences were assumed to be significant at *p* < 0.05.

## 3. Results

### 3.1. Characteristics of Study Objects

Among the 53 patients, the most common defects were tongue (*n* = 27, 50.9%), followed by composite defects such as mandibular or maxillary continuity defect with overlying facial skin defects, or hypopharyngeal defect with skin defect (*n* = 8, 15.1%). Accurate preoperative information on the vascular pedicle and perforators is helpful either in composite defects (*n* = 8) for chimeric design or in maxillary defects (*n* = 4) and in robot-assisted cases with retroauricular approach (*n* = 9) where sufficiently long vascular pedicle is preferred, summing up to 40% (*n* = 21) of the patients (Table 1). A total of 2 flaps out of 53 were failed including one late failure at the 9th postoperative day, resulting in a success rate of 51/53, 96.2%.

### 3.2. Predicting Presence of the Perforators with CTA

**Figure 2:**
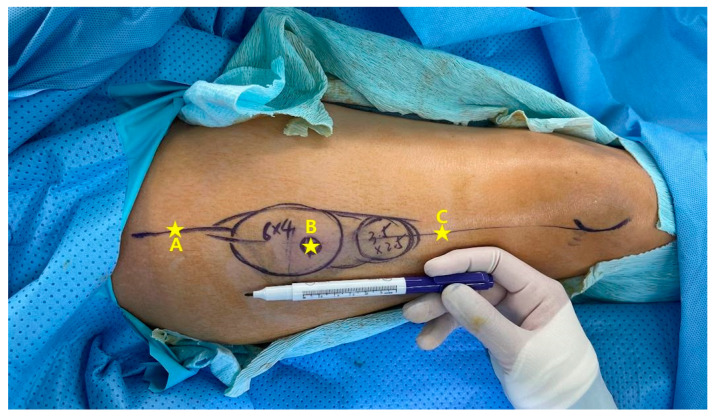
Three perforators A, B, and C were depicted in this patient’s angiography. Only the second perforator (B) was harvested with the skin paddle. The first perforator (A) was found, but was not harvested with the flap, and thus its presence was included in the analysis, but some of the characteristics such as its length were unable to be recorded and not included in the analysis. For the third perforator (C), its presence was unconfirmed as it was unexplored. Thus, the presence and the characteristics of it was unable to be recorded and was not included in the analysis.

Among the 85 intraoperatively found perforators, 79 were identified in CTA (positive predictive value (ppv) = 100%, sensitivity = 79/85, 92.9%). Six perforators unidentified in CTA were newly found intraoperatively (Table 2). Among the intraoperatively found perforators, only the ones included in the required skin paddle were dissected and harvested with the flap (Figure 2). Thus, the characteristics besides the presence of some of the undissected perforators could not be intraoperatively confirmed and are thus not reflected in the analyses.

### 3.3. Characteristic of Perforators—CTA Findings vs. Intraoperative Findings

Among the intraoperatively-found 85 perforators, 81 were dissected and harvested with the flap. The distance between the estimated location of the perforator based on CTA finding and the actual location of the perforator found intraoperatively was calculated for 65 perforators (actual location data lacking for the remaining 14 perforators). The median distance was 9.6 mm [IQR 5.6–14.2] at the skin. Of the 79 perforators detected in preoperative CTA, there were 48 musculocutaneous (MC) perforators and 31 septocutaneous (SC) perforators. However, in reality, there were more MC perforators than expected in the actual operation field (MC: *n* = 69, 81.2%, SC: *n* = 16, 18.8%) (Table 2). The intraoperative finding differed more at the course than at the originating branch compared to CTA. Among the total 79 predicted perforators, 52 were correctly predicted in terms of type. Of the remaining 27, 21 were expected to be septocutaneous perforators based on CT images, suggesting lower difficulty in dissection, but they turned out to be musculocutaneous perforators (Table 3).

### 3.4. Length of Vascular Pedicles

The length of the vascular pedicle was measured and recorded in 61 perforators and analyzed. The length was roughly expected by subtracting the image cut number of the location where the descending or oblique branch initiated from the image cut number where the perforator was expected to penetrate the fascia and run into the subcutaneous layer, and multiply it by 2 mm, which is the CT slice thickness. The actual length of the vascular pedicle was measured upon harvesting, i.e., after ligation of the vascular pedicle. Measurement was conducted after locating the flap on a flat table surface. The mean expected length in CTA was 120.6 ± 53.8 mm (*n* = 61), and the mean actual pedicle length was 107.5 ± 29.2 mm (*n* = 61). The expected average length in CTA and actual pedicle length was 144.6 ± 48.6 mm and 115.9 ± 27.0 mm (*n* = 41) for the descending branch. Likewise, they were 71.5 ± 20.1 mm in CTA and 89.8 ± 27.1 mm (*n* = 19) for the oblique branch and 70.0 mm and 102.0 mm (*n* = 1) for profunda femoris (*p* < 0.05) (Table 4). The length of pedicles tended to increase as the perforators were depicted distally.

**Figure 3:**
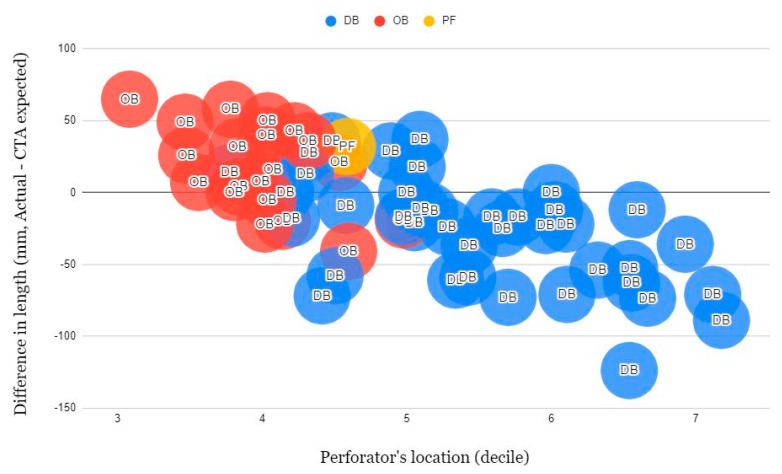
Zeropoint (0) indicates the anterior superior iliac spine and final point (10) indicates the superior-lateral point of the patella. Perforators depicted at CTA more proximal and originating from OB more tend to have actually longer pedicles than expected length and originating from DB and depicted at CTA more distal tend to have shorter pedicles than expected.

Regarding the discrepancy between expected and actual pedicle length, the more proximally located perforators tended to be longer than expected, and distally located perforators were shorter than expected (Figure 3).

## 4. Discussion

### 4.1. CTA for Safer, Easier ALT Free Flap Reconstruction with Optimal Outcome

**Figure 4:**
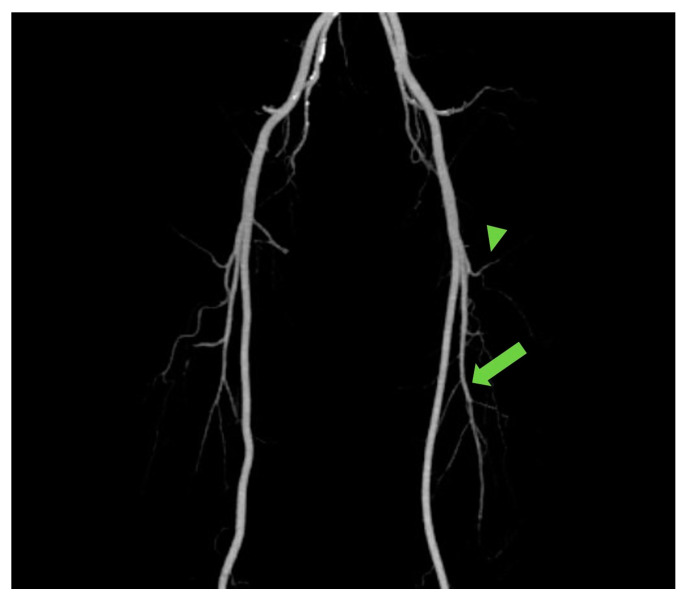
The maximum intensity projection (MIP) reconstruction showing the anatomy and course of perforators from transverse branch (arrowhead) and descending branches (arrow).

The ALT free flap is reliable and versatile owing to the appropriate diameter of its vascular pedicle and harvestability as a “freestyle flap”. In addition, it enables two-team approaches in the head and neck region, which can shorten the total operation time [8,9,10,11,12]. However, despite these advantages, there are clear drawbacks such as muscle-penetrating musculocutaneous perforators, and the varied branching pattern of LCFA, resulting in time-consuming dissection [13]. To overcome these hassles, the anatomical studies of branching patterns among ethnicities have been conducted [10,11,14], such knowledge regarding anatomic variation of the branching patterns potentially aiding planning and flap harvesting. However, with CTA, the branching pattern of each individual’s LCFA can be seen at a glance in maximum intensity projection (MIP) [15] (Figure 4). Moreover, CTA can provide individualized information regarding the location, number, length, and course of perforators in each patient, actually aiding in planning and designing the reconstruction and harvesting of the flap [6,16].

### 4.2. Course of the Perforators

Perforators of the ALT flap may show a septocutaneous course or may penetrate muscle. Choi et al. reported that musculocutaneous perforators prevail in the Korean population, as Wolff et al. and Zhou et al. reported generally [8,11,17,18]. The positive predictive value (ppv) for predicting the course of perforators in CTA was 89.4% for musculocutaneous perforators and 34.4% for septocutaneous perforators (Table 4), which implies a majority of perforators thought to be septocutaneous are actually musculocutaneous.

This misprediction seems to result from the presence of ‘semi-septo’ or ‘musculosepto’ cutaneous perforators [19,20]. When most of the course passes along the septum and through the muscle in some portion of its course, it is actually a musculocutaneous perforator, but may be seen as septocutaneous in CTA because the contrast-enhanced perforator in CTA tends to be more noticeable than the muscle. One study found that septocutaneous perforators are larger in size compared to musculocutaneous perforators, which explains musculo-septocutaneous perforators being mistaken for septocutaneous perforators [20]. Though one may need to keep in mind that septocutaneous perforators in CTA can actually be musculocutaneous or semi-septocutaneous, the actual course being different from CTA finding is not a big problem except for an increase in dissection time.

### 4.3. Branching Pattern of LCFA and Length of Vascular Pedicle

In 2007, Choi classified LCFA types into four categories according to the running of the descending branch, and in 2008, Wong defined as oblique those branches between the transverse and descending branches [8,21]. The length of the pedicle varies with each of these branches, increasing from the oblique to the descending branch. In addition, the more distal the perforator, the more likely it was to have a longer pedicle.

**Figure 5:**
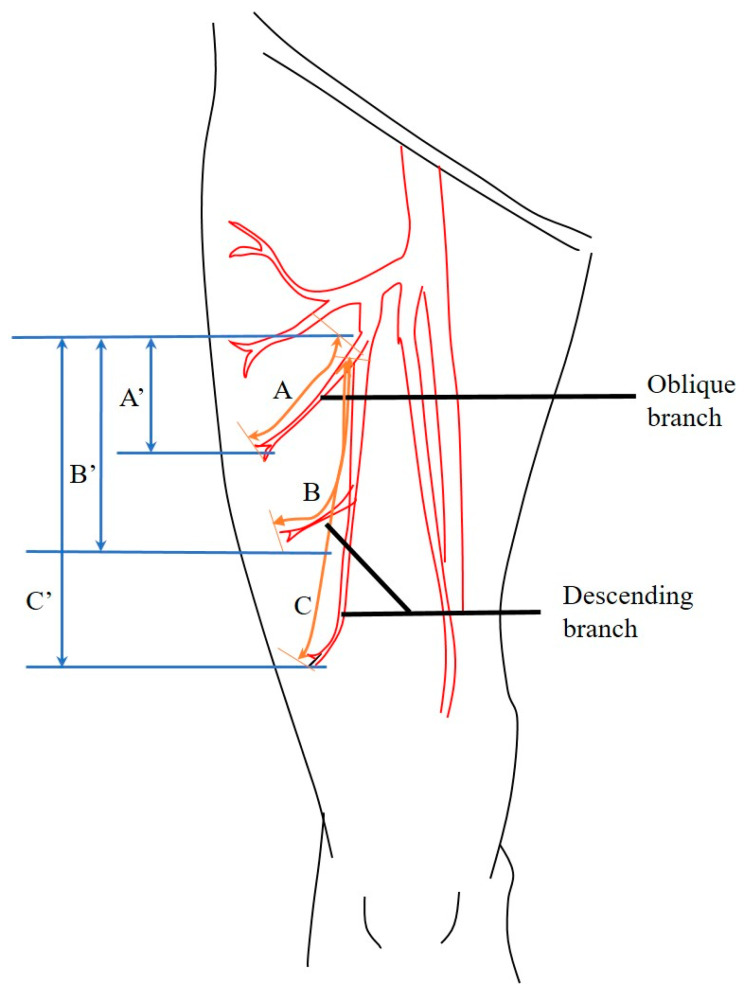
Actual length of pedicles harvested from the oblique and descending branches, and expected length based on CTA. The actual pedicle length is A for the oblique branch and B, C for the descending branch. The expected length was approximated as a straight line in CTA as A’ and B’, C’, from the branching point to the penetrating point. The actual length is thus bound to be shorter as the penetrating point is located distally even if both of them are from the same branch. (B-B’ > C-C’).

In practice, the perforators from the oblique branch were often longer than expected in CTA, while descending branches were shorter than expected. The perforator tended to be shorter than expected when located more distally. As the expected length is calculated by subtracting the image cut number where each branch initiates from the image cut number of a perforator penetrating into the subcutaneous layer, and multiplying it by CT slice thickness, the actual length of the oblique branch, a hypotenuse of a triangle, is unconditionally longer than the sides (Figure 5, A >> A’). As the perforators are located distally, or from the descending branch, they travel relatively straight caudally and their actual length tends to approximate the expected length (Figure 5, B ≥ B’ and C ≈ C’).

Using the number of CT slides to estimate pedicle length is primitive, but it is a simple way to intuitively estimate length. The information obtained in this way can be useful when preoperatively predicting the length of the vascular pedicle such as in free flap reconstructions of the maxilla or after robot-assisted retroauricular neck dissection, where a longer vascular pedicle is needed compared to the tongue, mandible, pharynx, or conventional transcervical approaches [22,23].

### 4.4. Location Discrepancy: CTA Expected vs. Intraoperative Finding

Yu reported that the distance between the actual perforators and Doppler-mapped ones varies depending on the type of Doppler or the location of the perforators and that the probability of the actual perforators being present within 10 mm from the point marked by Doppler may be 43~87% [24]. We expected a similar discrepancy in the case of CTA and, indeed, found a median 9.6 mm linear distance between the CTA-predicted location and the intraoperatively confirmed location. One reason for this difference may be the course of the perforator. As the perforators run obliquely closer to the skin, the point at which Doppler starts to be heard will gradually move away from the actual location of the perforators. The probability of the actual perforators being present within 10 mm of the location expected based on CTA was 51.6%.

### 4.5. Combining Doppler Flowmetry and CTA: How Beneficial Is It?

A large body of literature reports the use of color Doppler ultrasound (CDU) for perforator tracking in ALT. As Moore’s meta-analysis shows, CDU is a highly accurate device with a sensitivity of 95.3%. However, as the same analysis points out, it is not a convenient device to use [7]. In recent prospective studies evaluating the performance of CDUs in detecting perforators, all evaluations of perforators with CDUs were performed by “radiologists” and patients were taken to the ultrasound department for evaluation [5,25].

The CDU is a very large machine compared to a handheld Doppler device, and while it can be brought in when needed, it is not a convenient image for a non-radiologist surgeon to read. This inconvenience prevents surgeons from reacting to unforeseen situations by themselves such as when a last-minute change of plan is needed during surgery.

### 4.6. Am I the Only One Who Feels CTA to Be in Accurate?

In this study, CTA demonstrated a high ppv of 100% for predicting the presence of perforators. This indicates that if a perforator is visible in CTA, it is highly likely to be present in the expected location, although there may be some discrepancy in its exact location. Even more promising are the perforators that were not shown on CTA.

While CTA is known to show lower sensitivity for perforators smaller than 1.0 mm in diameter, the average diameter of the perforators at ALT is 0.85–0.9 mm [7,8], and perforators smaller than 0.5 mm also range from 22% to 31.9%, likely hard to see in CTA. This accounts for intraoperatively newly discovered perforators, not depicted in CTA [8,18].

In addition, most of the newly found perforators originated from the descending branch. These were usually located distally from the average location of the total perforators from the descending branch, the perforator diameter decreasing as it originated from a more distal branch, so this trend can be explained.

Our endeavor, while aiming for “more precise surgery” by utilizing advancements in technology, acknowledges the paradox of contradicting the pursuit of “simpler surgery” in the realm of surgical procedures where unpredictability is evident in an operation room. It is imperative to explore approaches that address such uncertainties, and with this intention, this study was conducted.

It is important to note that this research is retrospective and may have some missing or unrecorded data. A more comprehensive study could have been achieved by addressing these limitations.

## 5. Conclusions

In this study, a comparison was made between the appearance of perforators on CTA and the actual intraoperative findings. The perforators found in CTA were always present, while their course could be mispredicted to be septocutaneous. The more proximally located perforators tended to be longer than expected, and distally located perforators were shorter than expected. Knowing this information on when and how much the CTA can be inaccurate will aid in reconstructing complex defects.

## Figures and Tables

**Table 1:** Clinical characteristics of study objects.

Clinical Characteristics of Study Objects (*n* = 53)	
**Age (mean ± sd)**	57.5 ± 14.9
**Gender (*n*, %)**	
Male:Female	33 (62.3%):20 (37.7%)
**Type of defect and reconstruction (*n*, %)**	
Tongue	27 (50.9%)
Composite	8 (15.1%)
*Mandible + skin *	3
*Maxilla + skin*	2
*Mandible + total tongue + skin*	1
*Mandible + oropharynx*	1
*Hypophranx + skin*	1
Oropharynx	6 (11.3%)
Maxilla	4 (7.5%)
Buccal mucosa	4 (7.5%)
Mandible	2 (3.8%)
Hypopharynx	2 (3.8%)
**Type of approach used (*n*, %)**	
Conventional transcervical	50 (84.7%)
Robot-assisted retroauricular	9 (15.3%)
**Recipient artery (*n*, %)**	
Superior thyroid artery	40 (75.5%)
Facial artery	13 (24.5%)
**Recipient vein (*n*, %)**	
Tributaries of facial vein	28 (52.9%)
External jugular vein	26 (49.1%)
Internal jugular vein	1 (1.9%)

**Table 2:** The characteristics of perforators and flaps in patients with preoperative CTA.

	CTA Depicted	Intraoperative Findings
Number of detected perforators in one patient	79	85
4	0 (0.0%)	1 (1.9%)
3	3 (3.8%)	4 (7.5%)
2	20 (37.7%)	21 (39.6%)
1	30 (56.6%)	27 (50.9%)
Course		
Musculocutaneous	48 (60.8%)	69 (81.2%)
Septocutaneous	31 (39.2%)	16 (18.8%)
Origin of the perforator		
Descending branch	53 (67.2%)	58 (68.2%)
Oblique branch	25 (31.6%)	26 (30.6%)
Profunda femoris	1 (1.3%)	1 (1.2%)

**Table 3:** Predictive value of predicting the course of perforators.

		Actual Course Based on Intraoperative Finding (Number of Perforators)		
		Musculocutaneous Perforators	Septocutaneous Perforators	Total
Predicted course based on CTA finding (Number of perforators)	Musculocutaneous perforators	42	6	48
	Septocutaneous perforators	21	10	31
	Total	63	16	79

**Table 4:** Pedicle length for the branches.

	CTA Depicted	Intraoperative Findings
Mean length (mm, *n* = 61)	120.6 ± 53.8	107.5 ± 29.2
Descending branch (mm, *n* = 41)	144.6 ± 48.6	115.9 ± 27.0
Oblique branch (mm, *n* = 19)	71.5 ± 20.1	89.8 ± 27.1
Profunda femoris (mm, *n* = 1)	70.0	102.0

## Data Availability

Data is unavailable due to ethical restrictions.
